# Sex-specific difference for melanoma from immunotherapy advancement

**DOI:** 10.3389/fonc.2024.1484716

**Published:** 2024-11-14

**Authors:** Qianqian Li, Ying Dong, Yujiao Ma, You Mo, Jupeng Yuan, Xu Liu

**Affiliations:** ^1^ Shandong Provincial Key Laboratory of Precision Oncology, Shandong Cancer Hospital and Institute, Jinan, Shandong, China; ^2^ Shandong First Medical University and Shandong Academy of Medical Sciences, Jinan, Shandong, China; ^3^ Department of Radiation Oncology, Shandong University Cancer Center, Jinan, Shandong, China; ^4^ Laboratory of Molecular Cardiology, The First Affiliated Hospital of Shantou University Medical College, Shantou, Guangdong, China

**Keywords:** melanoma, sex, incidence, mortality, immunotherapy

## Abstract

**Background:**

The evaluation of melanoma incidence and mortality trends based on population characteristics, with a particular focus on sex differences, is of utmost importance.

**Methods:**

The gender-stratified analysis of melanoma mortality across various calendar years was conducted. Utilizing the Joinpoint software, we detected alterations in the incidence rates and delineated the mortality trends.

**Results:**

Melanoma’s incidence-based mortality exhibited a rising trajectory between 2005 and 2010, characterized by an annual percent change (APC) of 2.95%. However, there was a significant decrease in mortality from 2015 to 2019, with an APC of -4.39%. Notably, the mortality among men decreased by about 5.84% between 2015 and 2019, while there was no significant downward trend in the mortality rate among women. Subsequent analysis revealed no statistically significant variation in the 2-year survival rate of female patients aged 45-54 years among different age groups (Z=-0.775, p >0.1).

**Conclusions:**

Between 2015 and 2019, against the backdrop of stable melanoma incidence rates in the United States, there was a significant decline in mortality. Our analysis suggests that the utilization of immunotherapy may account for the observed reduction in mortality, with particularly notable benefits for male patients. However, female patients, especially younger women, did not derive significant advantages.

## 1 Introduction

Melanoma, a malignant tumor originates from melanocytes, representing a significant malignancy with substantial impact on public health (1, 2). Its remarkable invasiveness and high metastatic potential present a considerable clinical challenge. Melanoma ranks fifth among newly diagnosed cancer cases, accounting for approximately 4-6% (3). While the incidence trends of melanoma have been thoroughly documented, the mortality trends, particularly in relation to the impact of therapeutic innovations, remain less understood. Evaluating the mortality trends is of paramount importance, as the potential introduction of melanoma screening programs and advancements in treatment are anticipated to exert a range of influences on future mortality rates. This analysis is essential for informing public health strategies and optimizing melanoma management protocols. Furthermore, it is imperative to disaggregate the incidence-based mortality and survival rates by gender, given the distinct prevalence patterns of melanoma among different sexes (4–6). This stratification will provide a more nuanced understanding of the disease’s epidemiological profile and inform targeted interventions and research initiatives.

The treatment of melanoma has historically posed significant challenges, with limited efficacy observed in response to conventional chemotherapy regimens. In recent years, significant advancements have trans-formed the melanoma treatment arena, with the emergence of targeted therapies and immunotherapies profoundly altering the prognosis for patients. These innovative approaches have not only expanded the treatment options available but also improved the survival quality and overall survival for individuals afflicted with this aggressive form of skin cancer. The BARF mutation in melanoma serves as a paradigmatic illustration. Therapeutic agents designed to target cancers harboring BRAF mutations represented a pioneering class of targeted therapies for the treatment of advanced melanoma. Vemurafenib and dabrafenib, which selectively target the BRAF-V600E mutation, exert a negligible effect on the wild-type BRAF protein (7–9). The National Comprehensive Cancer Network (NCCN) advocates for BRAF mutation status testing in high-risk melanoma cases to inform clinical management strategies. The U.S. Food and Drug Administration (FDA) first approved an immune checkpoint inhibitor (ICI) therapy for melanoma in 2011, ipilimumab, which is an antibody that targets the cytotoxic T-lymphocyte-associated protein 4 (CTLA-4) (10). This approval was followed by the FDA’s endorsement of anti-programmed cell death protein 1 (PD-1) antibodies pembrolizumab and nivolumab in 2014, marking further advancements in immunotherapeutic options for melanoma treatment (11, 12). The therapeutic repertoire was further expanded in 2015 with the FDA approval of the combinatorial regimen of ipilimumab and nivolumab, heralding a new era in melanoma treatment strategies (13). However, a comprehensive evaluation of the impact of therapeutic advancements on melanoma’s population-level mortality remains elusive.

Employing the incidence-based mortality approach, this study delineates melanoma’s mortality trends in the United States from 2001 to 2019. The analysis incorporates gender-stratified data to assess the influence of incidence rates and melanoma-specific survival on mortality trends. This approach reveals distinct gender-related patterns and highlights disparities in melanoma incidence and patient outcomes.

## 2 Materials and methods

### 2.1 Study data

The Surveillance, Epidemiology, and End Results (SEER) program, a robust and exhaustive resource from the National Cancer Institute, provides data on cancer incidence and mortality across a significant segment of the U.S. population. Our research cohort was extracted from the SEER 17-registries database (SEER 17 Regs Research Data, November 2022, encompassing variable years from 2000 to 2020), with the dataset released in April 2023.

To ensure accurate categorization of melanoma subtypes, our investigation utilized the classification system proposed by Lewis et al, which applies the morphology codes from the International Classification of Dis-eases for Oncology, 3rd Edition (ICD-O-3) (14). Cases identified exclusively via death certificates or autopsy reports were excluded from our analysis due to the frequent absence of subtype-specific data. We computed the incidence and incidence-based mortality rates of melanoma for the timeframe from 2001 to 2019, incorporating adjustments for possible reporting lags and the repercussions of the COVID-19 pandemic on data collection (15).

### 2.2 Statistical analysis

In this investigation, we harnessed the SEER*Stat software (version 8.4.2) and Joinpoint software (version 5.0.1), both furnished by the National Cancer Institute, for the extraction and analysis of data. Our study’s emphasis was on the incidence of melanoma and its cancer-specific survival rates. The analytical process entailed calculating the ratio of documented melanoma-related fatalities to the standardized population size, utilizing this approach to gauge the influence of screening initiatives and therapeutic interventions on the mortality trends (16).

Our study presents a comprehensive analysis of melanoma incidence and incidence-based mortality rates, stratified by year and gender. We employed piecewise regression to delineate the temporal dynamics in age-standardized rates, disaggregated by sex. Utilizing the Joinpoint program, we discerned linear segments within each trend curve, with the corresponding percentages signifying the APC within the defined time spans. A marked asterisk (*) indicates instances where the APC deviates significantly from zero at the P < 0.05 level (17). Conclusively, we adopted a relative survival methodology to ascertain the 2-year and 5-year relative survival rates for melanoma patients, categorized according to their sex and the calendar year of diagnosis.

### 2.3 Ethics statement

The investigation was executed utilizing the SEER database, strictly adhering to the ethical guidelines set forth in the Declaration of Helsinki. We secured authorization for accessing the research data from the SEER program, and informed consent was waived due to the anonymized nature of the patient data.

## 3 Results

### 3.1 Trends in incidence and incidence-based mortality from melanoma

**Figure 1:**
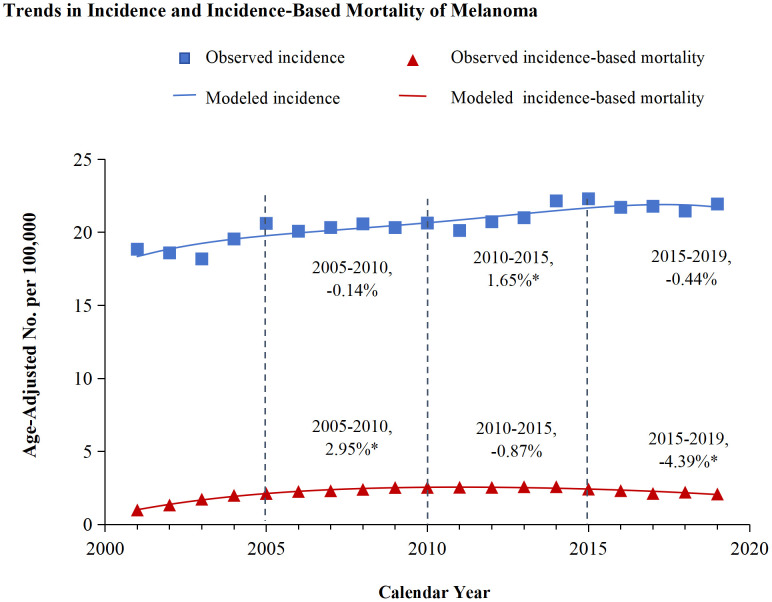
Trends in incidence and incidence-based mortality of melanoma. The figure presents the modeled trends in melanoma incidence (blue line) alongside the modeled trends in incidence-based mortality (red line). The incidence data are symbolized by blue squares, whereas the empirical incidence-based mortality data are represented by red triangles. Percentage values juxtaposed with the data points signify the annual percentage change across the defined time period. Annual percentage changes that are statistically distinct from zero (P < 0.05) are highlighted with asterisks.

**Figure 1** illustrated the analysis of melanoma incidence and incidence-based mortality from 2001 to 2019. The incidence of melanoma exhibited varying trends at different time periods. From 2005-2010, the annual incidence of melanoma exhibited a decline of 0.14%, which was not statistically significant. Similarly, from 2015 to 2019, the annual incidence decreased by 0.44% without statistical significance. However, the incidence exhibited a significant annual increase of 1.65% from 2010 to 2015. Melanoma incidence-based mortality showed promising trends from 2001 to 2019. Between 2005 and 2010, the incidence-based mortality for melanoma escalated by 2.25%. Subsequently, from 2010 to 2015, a downward trend in mortality was observed, which was not statistically significant. However, from 2015 to 2019, there was a significant annual drop of 4.39%.

### 3.2 Gender difference in incidence and incidence-based mortality of melanoma

**Figure 2:**
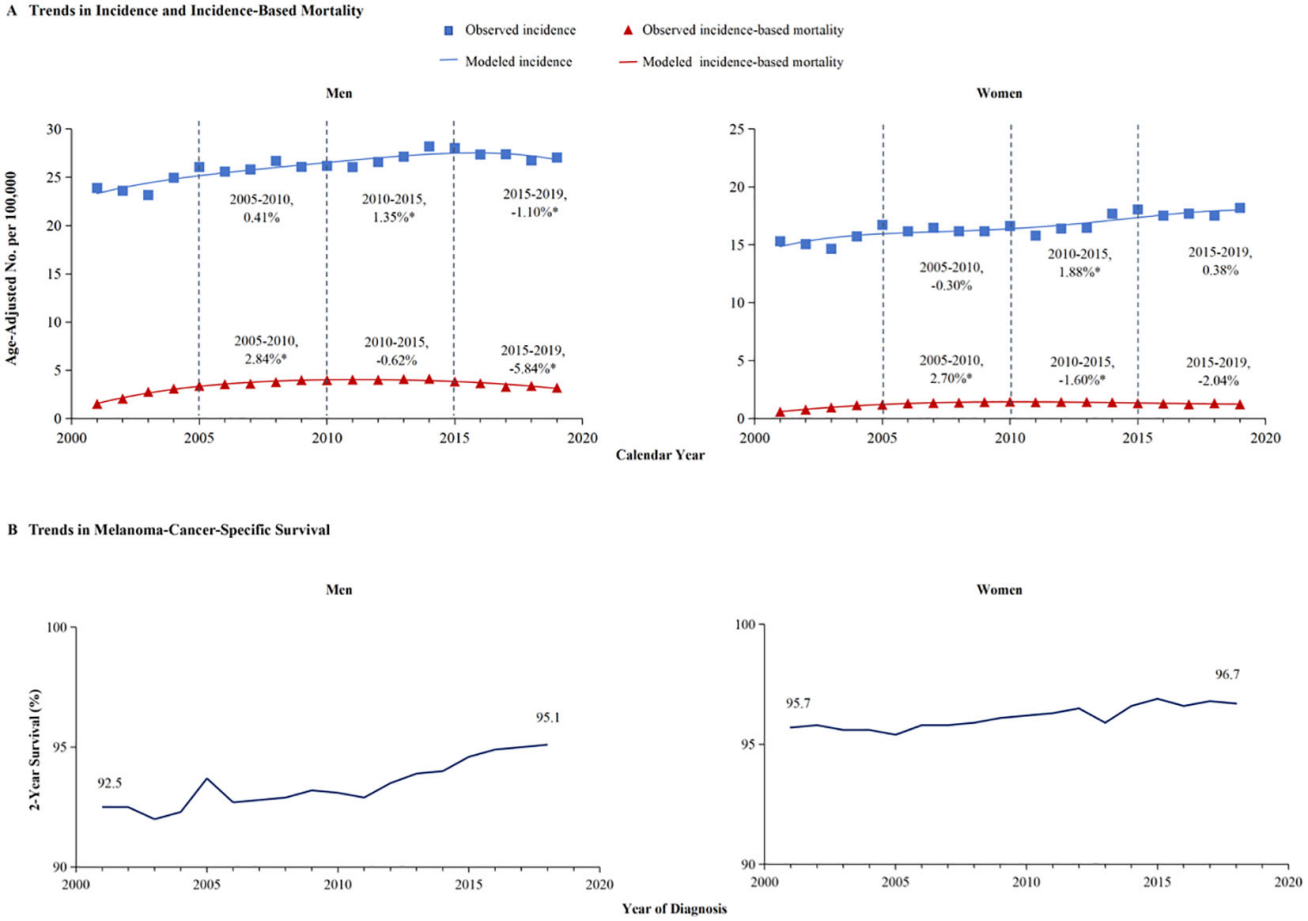
The incidence, incidence-based mortality, and survival trends of melanoma among men and women. **(A)** This panel displays the incidence (blue line) and incidence-based mortality (red line) trends for melanoma, stratified by gender. The data for incidence are symbolized with blue squares, and the corresponding incidence-based mortality data are marked with red triangles. **(B)** The 2-year cancer-specific survival, categorized by year of diagnosis, demonstrate significant improvement in men’s survival from 92.5% in 2001 to 95.1% in 2019, whereas women’s survival has increased from 95.7% to 96.7%.

A meticulous analysis of melanoma incidence and incidence-based mortality from 2001 to 2019, stratified by gender, is presented in **Figure 2**. The left panel of the figure illustrates the incidence-based mortality rates of melanoma among males. Consistent with the overall incidence-based mortality trend depicted in **Figure 1**, the mortality among male melanoma patients underwent an initial increase followed by a subsequent decline (**Figure 2A**). From 2005 to 2010, the incidence-based mortality escalated by 2.84%. Subsequently, between 2010 and 2015, a downward trend observed, which was not statistically significant. However, a statistically significant annual decline of 5.84% between 2015 and 2019. The 2-year relative survival rate for melanoma among male patients exhibited a marked improvement, escalating from 92.5% in 2001 to 95.1% by 2019, as depicted in **Figure 2B**. Conversely, the incidence of melanoma among females demonstrated stability from 2005 to 2010. Subsequently, a 1.88% annual increase was observed from 2010 to 2015, which plateaued, maintaining a consistent trend from 2015 to 2019.And, incidence-based mortality of women exhibited distinct patterns diverging from those observed in the general population and among men (**Figure 2A**). The incidence-based mortality in women increased by 2.70% from 2005 through 2010, followed by a significant decrease of 1.60% from 2010 to 2015. Between 2016 and 2019, the mortality dropped by 2.04% without significant statistical difference. The 2-year relative survival rate for women with a small increase from 95.7% in 2001 to 96.7% in 2019 (**Figure 2B**). The 2-year relative survival rates of men and women from 2001 to 2019 are further detailed in **Table 1**. The 2-year relative survival for men was 95.2% ± 0.1% in the period of 2005-2009, which increased to 95.8% ± 0.1% during 2010-2014, and then increased to 96.7% ± 0.1% in the period of 2015-2019. In comparison with the survival rate observed during 2010-2014, there was a significant improvement in the survival rate during 2015-2019 (Z =5.993, p <0.01). However, the survival rates between 2010-2014 (97.1% ± 0.1%) and 2015-2019 (97.5% ± 0.1%) showed no statistically significant difference among women (Z =1.649, p >0.1).

**Table 1:** 2-year relative survival rates of calendar year.

Cohort	Sex							
	Male				Female			
	No.	RS± SE (%)	Z value	P value	No.	RS± SE	Z-value	P value
Years of diagnosis								
2001-2004	25539	93.4 ± 0.2	Reference		20607	96.4 ± 0.2	Reference	
2005-2009	66121	95.2 ± 0.1	2.492	<0.01	29132	96.4 ± 0.1	-0.007	>0.1
2010-2014	72896	95.8 ± 0.1	5.613	<0.01	31646	97.1 ± 0.1	2.941	<0.01
2015-2019	81083	96.7 ± 0.10	10.834	<0.01	35445	97.5 ± 0.10	4.457	<0.01
2015-2019 Vs 2010-2014			5.993	<0.01			1.649	>0.1

**Figure 3:**
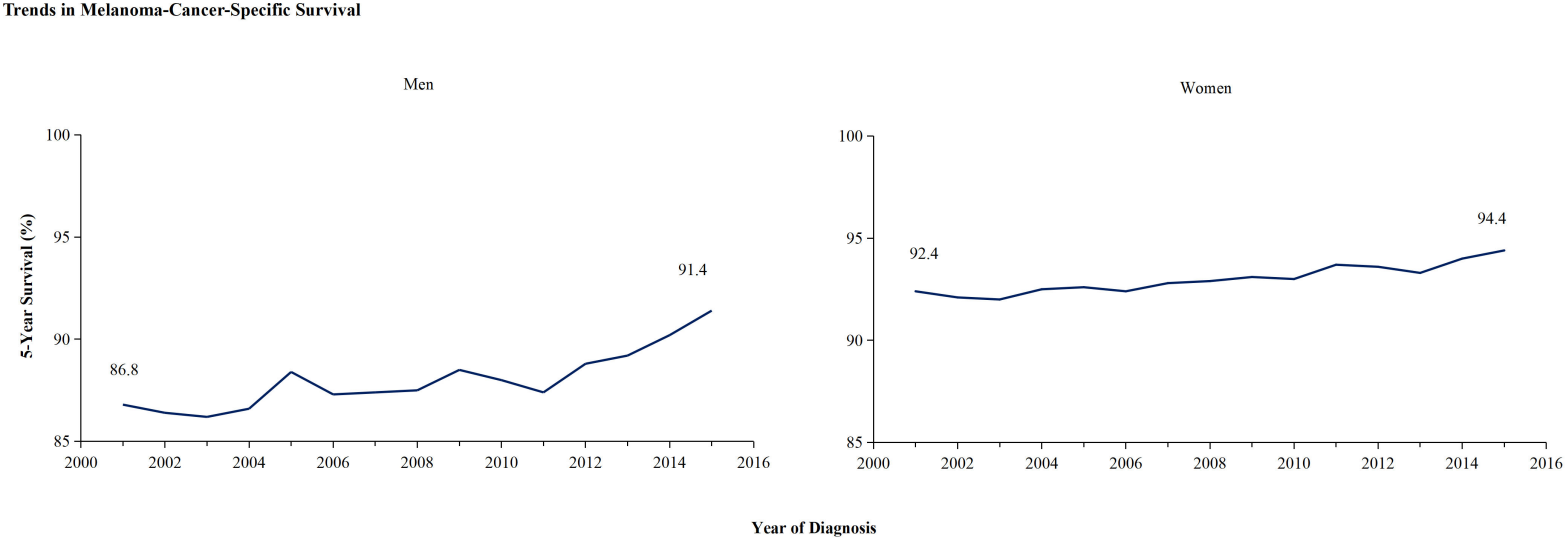
The 5-year cancer-specific survival trends of melanoma among men and women. The 5-year cancer-specific survival, categorized by year of diagnosis, demonstrate significant improvement in men’s survival from 86.8% in 2001 to 91.4% in 2015, whereas women’s survival has increased from 92.4% to 94.4%.

In light of the higher 2-year survival rate for melanoma, a further analysis on the 5-year melano-ma-cancer-specific survival in both men and women was conducted. At the most recent time point analyzed, which is 2015, the 5-year survival rate among female patients (96.3% ± 0.4%) was significantly higher than that among male (93.7 ± 0.5) (Z =4.716, p <0.01) (**Table 2**). **Figure 3A** illustrates that the male population exhibited a significantly elevated 5-year survival rate. The 5-year survival showed a significant increase from 86.8% in 2001 to 91.4% in 2015. Females demonstrated an upward trend in their 5-year melanoma-specific survival; however, this increase was less significant compared to that observed in males, mirroring the pattern seen in the 2-year survival. The 5-year melanoma-specific survival showed in women from 92.4% in 2001 to 94.4% in 2015 (**Figure 3B**). Following this trend, it is anticipated that the survival rates between male and female patients will further converge. Different patterns based on estimations of incidence and incidence-based mortality were observed between men and women. The observed decrease in incidence-based mortality exceeded that of the incidence rates themselves, underscoring the efficacy of targeted therapies and immunotherapies in improving outcomes among melanoma patients. The FDA granted approval to Vemurafenib in 2011 for the treatment of metastatic melanoma harboring BRAF-V600 E mutations. In the same year, Ipilimumab was initially approved by the FDA for ICI therapy in melanoma, marking a significant milestone in the treatment of this disease (11) which resulted in a significant decrease in incidence-based mortality compared to previous years. In 2015, the FDA granted approval for the combination therapy of Ipilimumab and Nivolumab to treat melanoma, thereby expanding treatment options and ushering in a new era of melanoma treatment strategy (13). The panel in trends of incidence-based mortality from male displayed the generation of combination therapy in 2015, as evidenced by a change in slope (**Figure 2A**). However, compared to male counterparts, female appear not to have derived significant benefits in their incidence-based mortality from novel therapeutic strategies, despite the incidence of melanoma remaining stable in this demographic.

**Table 2:** 5-year relative survival rates in male and Female.

2015	5-year relative survival			
	No. (%)	RS± SE (%)	Z value	P value
Sex				
Male and Female	16351	94.9 ± 0.3	Reference	
Male	9194	93.7 ± 0.5	-2.785	<0.01
Female	7157	96.3 ± 0.4	2.787	<0.01
Female vs Male			4.716	<0.01

### 3.3 Melanoma-cancer-specific survival across various age groups in men and women

**Figure 4:**
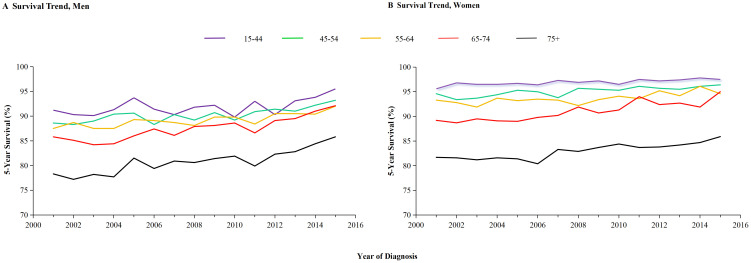
Survival trends of melanoma among men and women categorized by age groups. The 2-year melanoma -specific survival rate is presented by age groups in **(A, B)** for males and females, respectively.

Between 2001 and 2019, melanoma-specific survival improved for both men and women. However, given the varying impact across different age groups, postmenopausal women may experience changes in their survival outcomes. Consequently, we have estimated the differences in survival between men and women across various age groups. **Figure 4** illustrates that the improvement in 2-year melanoma-specific survival rates varies between males and females across different age groups. An enhancement in survival rates is observable in all age groups for males. However, among female patients, no significant survival benefits were noted in the younger age cohort, whereas a notable increase in survival rates was observed in females over the age of 55, as evidenced by the slope of the lines. The 2-year relative survival rates in different age from 2001 to 2019 are further detailed in **Table 3**. Between 2010 and 2014, there was a significant improvement in the 2-year relative survival rate across all age groups, with rates of 96.4% ± 0.2% (p <0.1) for individuals aged 45-54 years, 95.7% ± 0.2% (p <0.1) for those aged 55-64 years, 95.7% ± 0.2% (p <0.1) for those aged 65 to74 years, and finally, a rate of 93.7% ± 0.4% (p <0.1) for individuals aged 75 and older. However, during the period from 2015 to 2019, while the 45-54 age group reported the highest relative survival rate of 97.5% ± 0.2% (p <0.1) among all age groups, the trend did not demonstrate a statistically significant increase (p >0.1). In contrast, the 55-64 years maintained a survival rate of 96.6% ± 0.2% (p <0.1), the 65-74 age group was at 96.1% ± 0.2% (p <0.1), and patients over 75 years achieved a survival rate of 95.1% ± 0.3% (p <0.1), with all these trends showing significant enhancements. Statistical data indicate that the average age of menopause for female in U.S. is around 50 years old (18).

**Table 3:** 2-year relative survival rates in sex and age.

Cohort	2-year relative survival							
	2010-2014				2015-2019			
	No.	RS± SE (%)	Z value	P value	No.	RS± SE	Z value	P value
Age (Years)								
15-44	12739	97.5 ± 0.1	Reference		12228	97.6 ± 0.2	Reference	
45-54	13452	96.4 ± 0.2	-4.789	<0.01	12804	97.5 ± 0.2	-0.775	>0.1
55-64	17454	95.7 ± 0.2	-7.645	<0.01	20154	96.6 ± 0.2	-4.898	<0.01
65-74	15361	95.7 ± 0.2	-6.794	<0.01	20113	96.1 ± 0.2	-3.664	<0.01
75+	13731	93.7 ± 0.4	-9.237	<0.01	15640	95.1 ± 0.3	-6.72	<0.01

## 4 Discussion

In this investigation, we elucidate the mortality trends among melanoma patients, stratified by gender, while incorporating the dynamics of incidence and survival patterns. We have observed an improvement in the mortality since 2010, following the FDA approval of targeted therapies and immunotherapies. This improvement was particularly pronounced between 2015 and 2019, shortly after the recommendation of com-bination immunotherapy, with a significant reduction in incidence-based mortality, especially among male patients. However, no significant change was observed in the mortality rates among female patients. Significantly, within the female patient population, postmenopausal patients seemed to derive greater benefits from immunotherapy compared to their younger counterparts.

Melanoma prevention presents ongoing challenges, with epidemiological surveys revealing a substantial 320% escalation in melanoma incidence from 1975 to 2018 (19). Our research shows that there was no significant downward trend in the incidence of melanoma during the observed time period. Conversely, there was an increase in melanoma incidence from 2010 to 2015, which aligns with previous observations. The screening of individuals with risk factors and high-risk groups should be further strengthened to ensure a comprehensive evaluation.

During the preceding decade, the therapeutic domain for melanoma has undergone profound evolution. Specifically, melanoma incidence-based mortality was escalating prior to 2010. The introduction of targeted therapy and immunotherapy subsequently transformed the treatment landscape for advanced melanoma. Before these treatments, Dacarbazine stood as the sole FDA-approved chemotherapy agent for metastatic melanoma, yielding response rates between 7% and 12%, and a median overall survival (mOS) ranging from 5.6 to 7.8 months (8). A study published in N Engl J Med in 2010 showed that BRAF inhibitors effectively treat most patients with metastatic melanoma carrying the BRAF V600E mutation, leading to complete response or partial response (7). The BRIM-3 clinical trial substantiated that vemurafenib markedly enhanced OS and progression-free survival (PFS) in patients with untreated, metastatic melanoma harboring the BRAF V600E mutation, as compared to the use of dacarbazine (8). Furthermore, vemurafenib was designated the inaugural therapy for advanced melanoma to receive FDA approval in 2011. The advent of targeted therapy initiated a decline in the previously escalating melanoma mortality rates. Concurrently, the clinical deployment of immunotherapy has been transformative for the treatment paradigms of various cancers, notably with melanoma being among the pioneering neoplasms to benefit from such interventions. In 2011, the FDA sanctioned ipilimumab, a fully human monoclonal antibody, for metastatic melanoma therapy (10). Subsequently, a suite of agents directed against the PD-1/PD-L1 pathway, including nivolumab, pembrolizumab, and atezolizumab, have been authorized for melanoma treatment (12, 20, 21). Recognizing the potential for synergistic effects between distinct immunotherapeutic classes to activate diverse components of the immune response, clinical development advanced to encompass trials of combination immunotherapies. Clinical trials have demonstrated that combination immunotherapy significantly benefits melanoma patients, including the CheckMate-069 (22, 23) and CheckMate-067 trials (24–26). In 2015, the FDA approved the combination therapy of ipilimumab and nivolumab, further expanding treatment options and heralding a new era in melanoma treatment strategies (13). Our statistical results corroborate that the improvement in melanoma survival coincides with the introduction and approval of targeted therapies and immunotherapies. Given the nature of the study, we cannot dismiss the possibility that changes in other contributing factors may have influenced the declining melanoma mortality. While treatment methods can have an immediate effect on mortality, upstream factors such as changes in risk factors tend to exert a more gradual and diffuse influence. Therefore, it is less likely that any alterations in risk factors had an immediate impact on the observed rapid decline in mortality. However, caution must be exercised when attributing improved survival solely to immunotherapy therapies without data regarding their utilization among patients.

To our knowledge, this study presents the inaugural evidence of a marked sex-based disparity in the impact of immunotherapy advancements on melanoma, as measured by incidence-based mortality. The results revealed a significant downward trend in male mortality from 2015 to 2019, while female mortality exhibited stability. In terms of survival rates, there was a notable disparity between the two groups in 2000. The 2-year relative survival rate for male increased from 92.5% in 2001 to 95.1% in 2019, whereas the corresponding rate for female patients stood at 95.7% in 2001 and gradually rose to reach 96.7% after two decades. Despite being higher than that of male patients, the survival rates have further converged. And it indicated a greater benefit for male patients from innovative treatment strategies. A study in 2022 found that the baseline characteristics, peripheral inflammatory response, and treatment toxicity differ significantly between male and female patients with advanced melanoma under-going immunotherapy (27). More notably, our findings indicate that immunotherapy appears to be more beneficial for post-menopausal female patients. Moreover, another study’s findings raise a concern: a higher incidence of melanoma is observed in women during their early life stages, whereas men exhibit increased rates in later life (28). The findings suggest that estrogen and progestins might exert a significant influence on melanoma pathogenesis and its resistance to immunotherapy. Research indicates that sex-based immuno-logical variations, which affect both innate and adaptive immune responses, are shaped by sex hormones and the genetic profiles that differ between males and females (29–32).Early studies report female tumor immunogenicity is low, leads to lower the curative effect of immunotherapy (33). Our results hold implications for clinical practice and inform the direction of future research: primarily, the evaluation of immunotherapy strategies must consider gender as a potential risk and benefit factor, although additional studies are warranted to confirm this association and to elucidate the extent of gender’s influence on the efficacy of immunotherapies. Secondly, when designing new immunotherapy studies, it is crucial for researchers to ensure equal participation of both women and men in clinical trials. Lastly, special consideration should be given to women before and after menopause as they may exhibit different reactions to immune therapy.

Although the study provides valuable information, it is crucial to acknowledge its limitations. Firstly, the SEER database lacks comprehensive data on targeted therapy and immunotherapy. Secondly, the relatively short follow-up duration after immunotherapy in melanoma patients within the SEER database hinders obtaining additional long-term survival outcomes. Thirdly, the prognosis provided by the SEER database is limited to survival time and does not include analysis of objective response rate (ORR), disease control rates (DCR), PFS, and quality of life. Finally, it is important to emphasize that initially immunotherapy was approved for the treatment of patients with unresectable or metastatic melanoma. However, subsequent publication of clinical trial results has expanded the application of immunotherapy in the broader spectrum of melanoma treatment. Nevertheless, it should be noted that this study included all patients with melanoma, which may introduce a certain degree of confounding bias. Based on the findings from clinical studies, there is evidence supporting the positive impact of immunotherapy across majority stages of melanoma; however, further validation in real-world settings and longer follow-up periods are warranted.

Our study indicates that since 2015, although the incidence of melanoma has not decreased, the clinical application of targeted therapies and immunotherapies has led to a significant reduction in incidence-based mortality. Combination immunotherapy treatment strategies have yielded greater benefits for male patients, while female patients, particularly those who are young, have not experienced significant advantages. Consequently, the assessment of risks and benefits of immunotherapy strategies in clinical research should take into account the patients’ gender. Further confirmation of this finding is warranted through larger-scale trials.

## Data Availability

Publicly available datasets were analyzed in this study. This data can be found here: URL: https://seer.cancer.gov/.
